# Correlation Between the C-reactive Protein-to-Albumin Ratio and Severity of Coronary Artery Disease in Patients With Myocardial Infarction

**DOI:** 10.7759/cureus.89580

**Published:** 2025-08-07

**Authors:** Muhammad Jibran Hakak, Mir Mohammad

**Affiliations:** 1 Emergency Medicine, Queen Elizabeth Hospital, London, GBR; 2 Cardiology, Davao Medical School Foundation, Davao City, PHL; 3 Internal Medicine, Davao Medical School Foundation, Davao City, PHL

**Keywords:** albumin, artery, coronary, correlation, crp, disease, infarction, myocardial, severity

## Abstract

Background

Coronary artery disease (CAD) is a major global health issue, and its severity assessment via the C-reactive protein-to-albumin ratio (CAR) is cost-effective and simple. However, the correlation between the CAR and CAD severity in patients with myocardial infarction (MI) remains understudied in less developed countries. Consequently, this study was designed to assess the correlation between CAR levels and the CAD severity among MI patients.

Methods

This study with a cross-sectional study design was carried out among 265 patients with MI at Davao Medical School Foundation Hospital, Davao City, over one year (June 2023 to June 2024). Enrollment of the patients was performed via consecutive sampling based on established inclusion and exclusion criteria. A self-designed proforma was applied to gather data. Patients were categorized into non-severe (scores ≤50) and severe CAD (scores >50) categories according to the Gensini scoring system. IBM SPSS Statistics for Windows, Version 25 (Released 2017; IBM Corp., Armonk, New York, United States) was used for data analysis. The comparative statistics were performed between non-severe and severe CAD groups using independent t-tests and chi-square tests. The association between the CAR and CAD severity was examined through Pearson's correlation analysis. Linear regression modeling was used to determine the predictive ability of CAR for CAD severity. Statistical significance was defined as a p-value < 0.05.

Results

Among 265 patients, 172 (64.90%) had non-severe CAD while 93 (35.10%) had severe CAD. Patients with severe CAD exhibited a markedly higher mean CAR (17.85 ± 15.21) compared to those with non-severe CAD (4.28 ± 2.16). Statistically significant differences were noted in Gensini scores (p=0.001) and CAR values (p=0.001) between the two study cohorts. A strong positive relationship was observed between CAR and the severity of CAD (r=0.78, 95% CI=0.73 to 0.83, p < 0.001). Furthermore, regression model analysis confirmed the CAR as a notable determinant of the severity of CAD, with a 95% CI of 1.75-4.96 (p < 0.001), positive values of unstandardized coefficient (3.23) and standardized coefficient (0.78), and an R-squared value of 0.84.

Conclusions

The present study presented a substantial positive correlation between the CAR and the severity of CAD among patients with MI. An increased CAR was associated with higher CAD severity suggesting CAR's potential utility in clinical practice for identifying patients at high risk of CAD, enabling prompt intervention, and monitoring their response to treatment.

## Introduction

Coronary artery disease (CAD) is a major global health concern, causing significant morbidity and mortality. It occurs when coronary arteries narrow or block due to atherosclerosis, reducing blood flow to the heart and potentially leading to heart attack, heart failure, and sudden cardiac death [1]. CAD can be broadly classified into two subtypes: acute coronary syndrome (ACS) and chronic coronary syndrome. 

ACS is categorized into three additional well-defined subgroups: STEMI (ST-elevation myocardial infarction), NSTEMI (non-ST elevation myocardial infarction), and unstable angina. Acute myocardial infarction (AMI) is a severe manifestation of ACS and includes NSTEMI and STEMI [2].

According to the World Health Organization (WHO), cardiovascular diseases account for 43% of non-communicable disease deaths. CAD caused 17.9 million deaths in 2019, with most occurring in low- and middle-income countries. This figure is expected to increase to 23.6 million in 2030 [3,4]. In the Philippines, CAD is among the top three causes of mortality, with a prevalence rate of 17.50% [5]. While most CAD cases are preventable, suboptimal implementation of prevention strategies contributes to premature deaths in these countries [6]. 

The underlying mechanism of CAD involves atherosclerosis, a chronic inflammatory process that promotes the formation and advancement of atherosclerotic lesions in the coronary vasculature. This complex pathological cascade is organized by the interaction of immune cells, inflammatory mediators, molecular signals, and atherogenic lipoproteins, ultimately leading to stenosis and obstruction of the coronary arteries, compromising blood flow to the cardiac tissues [7,8].

Several inflammatory markers, such as C-reactive protein (CRP), serum albumin, interleukin-6 (IL-6), IL-1 variant, fibrinogen, tumor necrosis factor-alpha (TNF-α), and monocyte colony-stimulating factor, are utilized in clinical settings to identify inflammatory reactions [2,9,10]. CRP and serum albumin are the commonly used parameters, and several studies have shown a strong link between these inflammatory markers and the severity of CAD among myocardial infarction (MI) patients [11-13]. CRP is a pro-inflammatory biomarker that amplifies inflammatory responses in arterial walls, accelerating the formation and advancement of atherosclerotic lesions. In contrast, albumin exhibits anti-inflammatory properties, suppressing platelet activation and aggregation, thereby mitigating the risk of coronary artery narrowing [10,14,15]. Therefore, the CRP-to-albumin ratio (CAR) has emerged as a promising biomarker for assessing the severity of inflammation and consequently predicting the disease progression in CAD patients [16,17]. In the literature, several studies have revealed a significant correlation between elevated CAR and an increased risk of CAD, particularly in the acute phase. This association suggests that CAR may be a valuable tool for identifying patients at high risk of CAD and monitoring their response to treatment. However, the correlation between the CAR and the CAD severity in MI patients remains inadequately understood, especially in resource-constrained settings that are common in developing countries [18,19].

In the Philippines, research work investigating the relationship between CAR and CAD severity among MI patients is lacking. Thus, this study seeks to assess the correlation between CAR and CAD severity in individuals with acute myocardial infarction. By clarifying the significance of CAR in evaluating CAD severity, this study offers valuable insights that may guide clinical decision-making, shape healthcare policy, and direct future investigations, ultimately improving CAD prevention and treatment outcomes in high-risk individuals.

## Materials and methods

Study design and study population

This study with a cross-sectional design was carried out at Davao Medical School Foundation Hospital, Davao, Philippines, over one year (June 2023 to June 2024). A cohort of 265 patients diagnosed with AMI who had a complete record of findings of coronary angiography was enrolled via consecutive sampling to mitigate selection bias, based on predetermined inclusion and exclusion criteria. The OpenEpi's sample size calculator was used to determine the minimum sample size of 246 patients, based on a previously reported CAD prevalence of 17.50% (Sison et al.), with a 95% confidence level, a 5% margin of error, and 80% power [5]. However, the current study's final sample size of 265 patients exceeded this calculated minimum, ensuring adequate power to detect significant associations. Before the study initiation, institutional review board approval was acquired from the respective foundation, and informed consent was waived from all participating patients after elaboration of the objectives of the study.

Inclusion and exclusion criteria

This research encompassed both male and female patients over eighteen years old, with a thorough medical history of ACS verified via electrocardiography (ECG), cardiac biomarkers, and coronary angiography. In contrast, individuals with less than 18 years of age and those with a documented history of prior heart diseases (congenital heart disease, ischemic heart disease, or heart failure), cardiac intervention (percutaneous coronary intervention or coronary artery bypass graft surgery), chronic hepatic or renal disease, anemia, autoimmune disorders, active infections, malignancies, pregnancy, and recent surgical interventions (during the preceding three months) were not considered into the study to minimize the potential confounding effects of these conditions on the study's outcomes.

Primary outcomes and secondary objectives

This study's primary objective was to examine the association between CAR values and the severity of CAD, as measured by the Gensini scoring system. Additionally, two secondary objectives were also pursued: firstly, to compare CAR values between patients with severe and non-severe CAD, and secondly, to assess the predictive value of CAR levels for CAD severity, thereby exploring its potential as a prognostic indicator.

Assessment of study parameters

Serum CRP and albumin levels were measured from blood samples as per hospital standards at admission, with reference ranges of 0-10 mg/L for CRP and 3.5-5.0 g/dL for albumin. The CAR was calculated by dividing the CRP level (mg/L) by the albumin level (g/dL) [15].

The criteria established by the American Heart Association for diagnosis of MI were applied, which necessitated a convergence of prolonged chest pain (at least 30 minutes), characteristic ECG Configurations suggestive of MI, and elevated cardiac enzyme levels [2]. CAD was identified via coronary angiography, according to the lesion classification guidelines of the American College of Cardiology/American Heart Association. CAD was defined as significant stenosis (≥50% luminal diameter) in any major coronary artery. The Gensini scoring system which is a widely acknowledged angiographic grading system for evaluating CAD severity, was applied in the present study for the assessment of the CAD severity. The Gensini score calculation follows a well-established methodology consisting of three main steps. First, lesion severity is quantified based on the extent of stenosis. Next, a weighted score is assigned according to the lesion's location within the coronary arterial tree. Finally, the total score is computed by aggregating individual lesion scores. Patients were subsequently categorized into two groups based on their Gensini scores: those with scores ≤50 were classified as having non-severe CAD, while those with scores >50 were classified as having severe CAD, in accordance with established guidelines [2,4,20]. To minimize inter-observer variability in the angiographic assessment, two experienced cardiologists independently validated the Gensini score using a standardized protocol.

Data collection tool

A self-designed proforma was utilized for the data collection, which consisted of two sections. The first section documented comprehensive patient clinical history and physical assessment observations, comprising of demographic characteristics (age, sex) and conventional CAD risk factors like family history, hypertension, diabetes mellitus, dyslipidemia, and smoking status. The second section recorded the results of diagnostic tests, including cardiac biomarkers, CRP, serum albumin, ECG, and coronary angiography, all of which were performed during management.

Data analysis

Statistical analysis was conducted employing IBM SPSS Statistics for Windows, Version 25 (Released 2017; IBM Corp., Armonk, New York, United States). Categorical data was described using frequencies and percentages, whereas continuous data was presented as mean ± standard deviation. We used parametric statistical tests for data analysis, as the Shapiro-Wilk test indicated that our data conformed to a normal distribution. Group comparisons for qualitative and quantitative variables were made utilizing the chi-square test and independent t-test, respectively. The relationship between Gensini scores and CAR values was evaluated by applying Pearson's correlation analysis. Furthermore, the prognostic ability of CAR values for Gensini scores was examined via a linear regression analysis. P-value < 0.05 was set as significant.

## Results

Out of 265 patients, 172 (64.90%) had non-severe CAD, while 93 (35.10%) had severe CAD. The mean values (± standard deviation) for study variables were age (59.21 ± 19.50 years), Gensini score (47.65 ± 34.29), CRP (32.87 ± 30.79), serum albumin (3.71 ± 0.20), and CAR value (9.75 ± 8.35). 

Table 1 presents the demographic and clinical profile of the study population. It has also highlighted the significant differences between the non-severe CAD and severe CAD groups in several primary parameters such as Gensini scores, CRP levels, serum albumin levels, and CAR values (p < 0.05). Furthermore, while the prevalence of severe CAD was greater among patients with male sex, a familial history of CAD, high blood pressure, diabetes mellitus, lipid disorder, and smoking history, no significant disparities were detected in age or the distribution of these risk factors between the two groups (p > 0.05).

**Table 1:** Demographic and clinical profile of the study population CAR: C-reactive protein-to-albumin ratio; CAD: coronary artery disease; SD: standard deviation p-values with the sign of (*) are of independent t-tests while p-values with the sign of (+) are of the chi-square test.

Variables N=265		Presentation of Variables	Severity Status of CAD		Independent t-test/ Chi-Square test	
					Test Statistics	
			Non-CAD Group n=172 (64.90%)	Severe CAD Group n=93 (35.10%)	t-value for Independent-test/ χ²-value for Chi-Square test	p-values
Age (Years) (Means ± SD)		59.21±19.50	58.10±15.02	60.14±18.79	0.90^*^	0.12^*^
Gensini Score (Means ± SD)		47.65 ±34.29	27.86±15.39	71.58±34.25	12.37^*^	0.001^*^
C-reactive Protein Level (mg/dL) (Means ± SD)		32.87 ± 30.79	18.41 ± 11.34	64.12 ± 52.48	9.24^*^	0.001^*^
Serum Albumin Level (g/dL) (Means ± SD)		3.71 ± 0.20	4.12 ± 0.14	3.59 ± 0.08	3.71^*^	0.001^*^
CAR Value (Means ± SD)		9.75±8.35	4.28±2.16	17.85±15.21	9.41^*^	0.001^*^
Gender	Male n (%)	160 (60.37)	104 (60.46)	56 (60.21)	0.70^+^	0.79^+^
	Female n (%)	105 (39.63)	68 (39.54)	37 (39.79)		
Family History of Coronary Disease	Yes n (%)	85 (32.10)	37 (21.52)	48 (51.61)	3.34^+^	0.07^+^
	No n (%)	180 (67.90)	135 (78.48)	45 (48.39)		
Hypertension	Yes n (%)	188 (70.94)	125 (72.68)	63 (67.74)	0.45^+^	0.52^+^
	No n (%)	77 (29.06)	47 (27.32)	30 (32.26)		
Diabetes Mellitus	Yes n (%)	148 (55.84)	99 (57.55)	49 (52.68)	0.12^+^	0.78^+^
	No n (%)	117 (44.16)	73 (42.45)	44 (47.32)		
Dyslipidemia	Yes n (%)	135 (50.93)	87 (50.58)	48 (51.61)	0.10^+^	0.82^+^
	No n (%)	130 (49.07)	85 (49.42)	45 (48.39)		
History of Smoking	Yes n (%)	112 (42.26)	53 (30.81)	59 (63.44)	3.27^+^	0.06^+^
	No n (%)	153 (57.74)	119 (69.19)	34 (36.56)		

Table 2 shows a significant positive relationship between CAR values and CAD severity, as indicated by Pearson's correlation test, in the study population. This notable relationship suggests that as Gensini scores increase, CAR values also tend to increase, indicating a direct association between CAD severity and CAR values. 

**Table 2:** Correlation between C-reactive protein-to-albumin ratio values and the coronary artery disease severity CAD: Coronary artery disease; CAR: C-reactive protein-to-albumin ratio.

Variables of Patients with Myocardial Infarction ( N=265)	Severity Status of CAD		Independent t-test		Pearson’s Correlation		
			Test Statistics		Test Statistics		
	Non-severe Coronary Artery Disease Group	Severe Coronary Artery Disease Group	t-value	p-value	Correlation Coefficient (r)	95% CI	p-value
Gensini score	27.86±15.39	71.58±34.28	12.37	0.001	0.78	0.73 to 0.83	0.001
CAR values	4.28±2.16	17.85±15.21	9.41	0.001			

Table 3 indicates that the simple linear regression analysis demonstrated a significant fit (R² = 0.84, p= 0.000), revealing a statistically significant direct association between CAR values and Gensini scores. The positive beta coefficient indicates that higher CAR values are significantly associated with elevated Gensini scores, suggesting a greater CAD severity.

**Table 3:** Evaluation of the predictive ability of C-reactive protein-to-albumin ratio value for coronary artery disease severity using a simple linear regression model. CAR: C-reactive protein-to-albumin ratio.

Variable		Test Statistics for Simple Linear Regression Model					
	Unstandardized Coefficient		Standardized Coefficient	95% CI	p-value	R^2 ^value	p-value of the F test
CAR values	3.23		0.78	1.75 to 4.96	0.001	0.84 (84.00%)	0.000

## Discussion

CAD is one of the major causes of mortality around the globe. Its timely diagnosis and risk stratification, followed by intervention, could significantly reduce the mortality related to it. Along with different advanced cardiac investigations, new inflammatory markers have been emerging that are cost-effective, straightforward, and easy to measure. These markers are especially useful in settings where advanced cardiac investigations are not readily available. One such marker is the CAR, which has shown great potential in assessing CAD severity and predicting outcomes. By applying such markers, healthcare providers can improve patient care and outcomes, particularly in resource-constrained environments [1,11]. In the current study, we have obtained important findings about the correlation between the CAR and the severity of CAD. Moreover, we determined the frequency of several conventional contributing parameters associated with CAD along with the differences in the spread of these predisposing components between two well-defined study groups: patients with non-severe CAD versus severe CAD. 

From the total present research population of 265 patients, 172 (64.90%) had non-severe coronary artery CAD, while 93 (35.10%) had severe CAD. A similar prevalence of severe and non-severe CAD was reported in a Pakistani study [4], whereas an Indian study has presented a higher frequency of severe CAD in its population [21]. The variation in CAD severity distribution between studies might be due to differing rates of classic cardiovascular risk factors in the respective patient groups. 

Evaluation of the study population's demographic profile showed that patients with severe CAD had a mean age of 60.14 years (SD±18.79), which was greater than that of patients with non-severe CAD (58.10 years, SD±15.02). Additionally, CAD was more common among male patients, representing 160 (60.37%) patients. These results are consistent with previous studies that observed similar demographic patterns in CAD patients [10,13].

Even though the frequency of severe coronary artery disease was greater among patients with a familial history of CAD, high blood pressure, diabetes mellitus, elevated lipid levels, and smoking history, however, the variation in the frequency was not statistically significant in contrast to those lacking these contributors. In the study cohort, hypertension was the most prevalent classic contributing variable, followed by diabetes mellitus, dyslipidemia, smoking history, and family history of CAD. This trend of these determinants, as reported by the present study, is consistent with the findings from several past studies [2,4,7,8].

This study revealed a strong and significant association between CAR values and CAD severity, where patients with more severe CAD had higher CAR values. This key finding aligns with numerous prior studies worldwide. A study from Egypt showed that CAR is a notable and cost-effective indicator of CAD severity [11]. A Chinese study also emphasized the link between elevated CAR values and increased CAD severity among myocardial infarction patients, noting that high CAR values predict poor outcomes in CAD patients [12]. A comparable study yielded analogous results about the relationship between CAR values and CAD severity, demonstrating that increased serum CAR values correspond to heightened CAD severity [13]. An Indian investigation also recorded similar Insights, showing that increased serum CAR values are correlated with elevated CAD severity [15]. In agreement with these observations, another study confirmed a significant role of CAR values in cardiovascular disease severity monitoring, echoing the results of this study [16]. Furthermore, a study from Taiwan also supported the findings of the current study that CAR can serve as a novel marker of coronary artery lesions [17]. A study from Greece also noted variations in CAR values across different patient groups with varying CAD severities [18]. Additionally, another study validated the findings of this investigation [19]. The results of this analysis support the use of the CAR as a novel biomarker for evaluating CAD severity in MI patients, consistent with several previous research conclusions.

The potential underlying mechanism linking CRP and CAD likely involves inflammatory processes, a crucial factor in atherosclerosis, and consequently, CAD advancement. As a positive acute-phase protein, CRP exacerbates arterial wall inflammation, hastening the development of atherosclerotic lesions. Conversely, as a negative acute-phase protein, albumin's anti-inflammatory effects inhibit platelet activation and aggregation, thereby reducing coronary artery constriction. Due to their contrasting roles in inflammation, which is a key aspect of CAD pathology, the CAR may be a trustworthy indicator of disease severity in myocardial infarction patients [9,10,14].

The findings of the current study have substantial clinical implications. The CAR value could serve as a valuable tool for the stratification of patients, particularly in resource-constrained environments where advanced cardiac diagnostic facilities are scarce. Utilizing the CAR as a biomarker for CAD severity may facilitate early identification and intervention in high-risk patients, potentially decreasing morbidity and mortality rates. By integrating the CAR in clinical practice, healthcare providers can make better-informed decisions, optimize patient care, and improve outcomes. Additionally, the simplicity and cost-effectiveness of CAR testing make it an attractive option for widespread adoption in various clinical settings.

Despite excluding individuals with pre-existing chronic conditions to reduce their impact on the relationship between CAR values and CAD severity, this study has several limitations. The single-center design may limit the generalizability of the findings to larger populations. The cross-sectional design prevents determining a causal relationship between CAR values and CAD severity and data of the outcomes of CAD among MI patients. Moreover, no adjustment of some potential confounders in the study, single measurement of CAR and albumin levels instead of serial calculation, and no calculation of any specific cut-off values of the CAR for the detection of severity of CAD might have influenced the results and their use in clinical practice. Thus, further studies using different designs (multicenter prospective cohort studies and interventional studies using CAR-guided therapy) conducted in various geographic locations are needed to confirm these results, address the limitations of the present study, and clarify the mechanisms underlying the association between CAR values and CAD severity.

## Conclusions

This study presented a strong positive correlation between CAR values and CAD severity among MI patients. Given its simplicity and cost-effectiveness, the CAR may be a valuable tool for cardiac risk assessment and determination of CAD severity among MI patients, particularly in resource-limited settings. Higher CAR values were correlated with more severe CAD, as reflected by higher Gensini scores, highlighting the importance of managing MI patients with high CAR values promptly, as they're more likely to have severe CAD. Thus, integrating the CAR into clinical practice may improve patient outcomes and reduce mortality rates. Further multicenter prospective studies and interventional studies using CAR-guided therapy are needed to confirm our findings and establish the clinical utility of the CAR. Overall, this study contributes to the growing body of evidence supporting the use of the CAR as a biomarker for CAD severity assessment among MI patients.

## Appendices

**Table 4:** Research proforma

Self-designed Research Proforma for Correlation Between C-reactive Protein-to-Albumin Ratio and Severity of Coronary Artery Disease in Patients with Myocardial Infarction			
Sections	Research Questions	Options: write/tick the option	
Section A (History, Physical Examination, and Medical Records)			
1. What was the age of the patient? (Years)			
2. What was the gender of the patient?		Male	Female
3. What were the presenting complaints of the patient?			
4. What was the duration of presenting complaints? (Minutes)			
5. Presence of family history of Coronary Artery Disease?		Yes	No
6. Past history of Diabetes Mellitus?		Yes	No
7. Past history of Hypertension?		Yes	No
8. Past history of Dyslipidemia?		Yes	No
9. Past history of smoking?		Yes	No
10. Past history of coronary artery disease/cardiac or any other Surgery?		Yes	No
11. Past treatment history?		Yes	No
12. Presence of any chronic disease other than those mentioned above?		Yes	No
13. What were the physical examination findings of the patient? (Vitals, General, and Systemic)			
14. What were the findings of the past Medical Record?			
Section B. (Investigations Reports)			
1. Electrocardiogram Findings			
2. Cardiac Biomarker Levels (Troponin I) (ng/ml)			
3. Coronary Angiography Findings			
4.Gensini Score			
5. Severity of Coronary Artery Disease based on Gensini Score	Non-severe Coronary Artery Disease Group (Up to 50)	Severe Coronary Artery Disease Group (Above 50)	
6. Serum C-reactive Protein Levels (mg/L)			
7. Serum Albumin Levels (g/dL)			
8. C-reactive Protein-to-Albumin Ratio Values (CAR)			
9. Serum Lipid Level (mg/dL)			
10. Lipid Level Status?	Normal (Less than 200 mg/dL)	Abnormal (Above 200 mg/dL)	
11. Was there any abnormality in the liver function tests/renal function test/C-reactive protein/erythrocyte sedimentation rate/white blood cell count?	Yes	No	
